# Reply to Taufiq and Pascoal, “Clarifying AI-attributable diagnostic gain across separately prepared slides in Techcyte-assisted coproparasitology”

**DOI:** 10.1128/jcm.01110-26

**Published:** 2026-10-27

**Authors:** Carles Rubio Maturana, Elena Sulleiro, Francesc Zarzuela, Patricia Martínez-Vallejo, Alejandro Mediavilla, Aroa Silgado, Carlos Turró, Martha Balladares, Ana Gracia, Carmen Paz, Dani García-Vega, Sol María San José-Villar, Albert Blanco-Grau, Fernando Moreno, Nieves Larrosa, Lidia Goterris

**Affiliations:** 1 Microbiology Department, Vall d’Hebron University Hospital, Vall d’Hebron Research Institute (VHIR) 203275, Barcelona, Spain; 2 Department of Microbiology and Genetics, Universitat Autònoma de Barcelona (UAB) 196650, Barcelona, Spain; 3 CIBERINFEC, ISCIII-CIBER de Enfermedades Infecciosas, Instituto de Salud Carlos III 38176, Madrid, Spain; 4 Clinical Biochemistry Department, Vall d'Hebron University Hospital 16810 , Barcelona, Spain; 5 Clinical Biochemistry, Drug Delivery and Therapy Research Group, Vall d'Hebrón Research Institute 203275, Barcelona, Spain; Mayo Clinic Minnesota, Rochester, Minnesota, USA

## REPLY

We sincerely thank Dr. Taufiq and Dr. Pascoal (1) for their interest in our study and for their thoughtful and constructive comments regarding our article, *Performance of the Techcyte AI-based image analysis system for coproparasitological diagnosis in clinical stool specimens: a retrospective evaluation study* (2). We appreciate the opportunity to further discuss and clarify the rationale behind our interpretation of the results.

Our laboratory has relied on conventional microscopy as the reference method for parasitological diagnosis for many years. Consequently, we have great respect for the work and expertise of microscopists, whose role remains fundamental in the diagnosis of parasitic diseases. At the same time, we believe that artificial intelligence and laboratory automation will become essential support tools for microscopy in the coming years. This perspective motivated our use of the term *diagnostic gain*, which was intended to reflect the additional value that AI-assisted systems may provide in a real-world clinical setting rather than to attribute all additional detections exclusively to the algorithm itself. Moreover, the term was properly defined in Materials and Methods section.

We agree that one of the main limitations of our study is its retrospective design, which we acknowledged in our publication. This approach makes it difficult to perform a direct comparison using the exact same slide for both methodologies, as the samples had already been processed according to the routine workflow of our laboratory. Furthermore, the Techcyte protocol requires the use of a 15 µL aliquot, and this relatively small volume may dry rapidly during the scanning process, potentially affecting slide quality and, consequently, the final results. For this reason, we decided to prepare independent slides from the same concentrated stool sample for each method, while fully acknowledging the limitations and potential sources of variability associated with this approach. Interestingly, despite the fact that conventional microscopy evaluated a larger sample volume, the AI-assisted automated system identified additional parasitic etiologies in several cases. This observation suggests that differences in diagnostic performance cannot be explained solely by the amount of material analyzed and that the ability of the system to localize structures of interest may also contribute to the observed differences.

Importantly, we also recognize that some of the additional detections may correspond to parasites present at very low burdens, for which their distribution across separately prepared slides can be stochastic. This phenomenon was observed in our reproducibility analysis for helminths with low parasite burdens and supports the possibility that a parasite may not be consistently represented in every preparation obtained from the same concentrated stool sample. From a clinical perspective, this also highlights an important consideration when using Techcyte: given that each preparation contains only 15 µL of concentrated material, analyzing multiple preparations may be warranted when there is a high clinical suspicion of intestinal parasitosis, particularly in cases with a potentially low parasite burden, in order to increase the probability of detection.

However, we believe that our findings remain clinically relevant. The use of the term *diagnostic gain* in no way invalidates our results or the scientific reliability of the article. By defining it clearly and presenting it in the results section, we believe it is legitimate and scientifically correct. Furthermore, the article has undergone rigorous peer review by the editors and reviewers of a prestigious international journal such as the Journal of Clinical Microbiology, who have accepted this terminology as the global scientific community.

We would also like to emphasize an important aspect of the Techcyte workflow that should be considered when interpreting our results. The AI algorithm is not designed to provide a fully autonomous diagnosis. Instead, the system requires mandatory expert review because the algorithm may identify artifacts, cellular debris, or other non-parasitic structures as potential objects of interest. Therefore, the diagnostic performance reported in our study should be understood as the result of a collaborative model combining artificial intelligence with expert microscopic interpretation rather than as an isolated evaluation of the algorithm itself.

Finally, we agree that future prospective studies should incorporate more standardized experimental designs, and such studies will undoubtedly help to better determine the extent to which the observed increase in diagnostic performance can be specifically attributed to the AI algorithm. Nevertheless, we believe that our study accurately reflects the potential of AI-assisted digital microscopy as a complementary tool capable of improving coproparasitological diagnosis in routine clinical practice.
